# Vortioxetine *versus* SSRI/SNRI with Pregabalin Augmentation in Treatment-Resistant Burning Mouth Syndrome: A Prospective Clinical Trial

**DOI:** 10.2174/1570159X22999240729103717

**Published:** 2024-07-29

**Authors:** Daniela Adamo, Federica Canfora, Giuseppe Pecoraro, Stefania Leuci, Noemi Coppola, Gaetano Marenzi, Giulia Ottaviani, Katia Rupel, Luca Pellegrini, Massimo Aria, Luca D’Aniello, Michele Davide Mignogna, Umberto Albert

**Affiliations:** 1 Department of Neuroscience, Reproductive Sciences and Dentistry, University of Naples Federico II, 5 *Via* Pansini, 80131, Naples, Italy;; 2 Department of Surgical, Medical and Health Sciences, University of Trieste, 447 Strada di Fiume, 34149, Trieste, Italy;; 3 Department of Biomedical and Neuromotor Sciences, University of Bologna, Viale Carlo Pepoli, 5, 40123, Bologna (BO), Italy; Highly Specialized Service for OCD and BDD, Hertfordshire Partnership University NHS Foundation Trust and University of Hertfordshire, Hatfield, United Kingdom;; 4 Department of Economics and Statistics, University of Naples Federico II, 21 *Via* Cintia, 80126, Naples, Italy;; 5 Department of Social Sciences, University of Naples Federico II, 1 Vico Monte della Pietà, 80138, Naples, Italy

**Keywords:** Burning mouth syndrome, vortioxetine, pregabalin, pain, anxiety, depression

## Abstract

**Objectives:**

The treatment of Burning Mouth Syndrome (BMS) represents a challenge in tailoring appropriate medication for individual patients. The augmentation of pregabalin to conventional treatment has shown promising outcomes in relieving pain and improving the quality of life in chronic pain conditions. This study aimed to compare the efficacy of vortioxetine with other antidepressants (SSRIs/SNRIs) in combination with pregabalin in a cohort of unresponsive BMS patients and to predict treatment response by using clinical data.

**Methods:**

A 52-week randomized, open-label, comparative clinical study was conducted, enrolling 203 BMS patients previously treated with one antidepressant for 12 weeks and non-responders to the treatment (clinical trial registration: NCT06025474). The study sample included two groups: Group A (136) received vortioxetine, while Group B (67) received SSRIs/SNRIs. Pregabalin (75 mg/day) was added to both groups, with a potential dosage increase to 150 mg/day for inadequate responders after 12 weeks. Treatment response was assessed with VAS and SF-MPQ, HAM-A, and HAM-D scores at 12, 24, 36, and 52 weeks. Stepwise logistic regression analysis was used to predict treatment response.

**Results:**

A total of 84 (61.8%) BMS patients in Group A and 39 (58.2%) in Group B showed treatment response. Group A reported a faster onset of action compared to Group B (44.8% *versus* 22.4% at time 1; *p*:0.002**) and lower adverse event rates (8.8% *versus* 20.8%; *p*:0.001).

**Conclusion:**

The addition of pregabalin to vortioxetine may be considered a potential treatment option for BMS. Further research is required to corroborate these findings and optimize personalized treatment approaches for BMS patients.

**Clinical Trial Registration Number:**

ClinicalTrials.gov (NCT06025474).

## 1 INTRODUCTION

Burning mouth syndrome (BMS) is an idiopathic chronic orofacial pain disorder characterized by a burning/dysesthetic sensation in the mouth lasting for more than three months without any discernible local or systemic pathological changes [1, 2]. It affects approximately 1.73% of the global population, with a higher prevalence observed among middle-aged or older women [2]. Beyond the burning sensation, individuals with BMS may also experience a range of oral and extraoral symptoms, including xerostomia, altered taste perception, metallic taste, mouth soreness, itching, and globus [3]. Additional symptoms such as vulvodynia, ophthalmodynia, and tinnitus can also occur [4, 5]. This complex symptomatology can significantly interfere with daily activities, such as eating, speaking, and sleeping, severely impacting social interactions, personal relationships, and work productivity, thereby impairing psychological well-being and overall quality of life [6-8].

The interconnection between pain and psychological distress is notable, with chronic pain patients at risk of developing long-term anxiety and depression and *vice versa*. Previous studies have reported a high prevalence of anxiety, depression, and sleep disturbances among BMS patients, along with cognitive decline [9-14]. To date, BMS management has primarily focused on improving patients' pain tolerance, often using clonazepam, classified as a benzodiazepine, which is recognized for its anticonvulsant or antiepileptic properties.

However, a more holistic approach, treating the individual rather than just the disease, is advocated to address both the psychological and social comorbidities frequently associated with BMS [13, 15]. This approach could mitigate symptom severity and enhance the quality of life for those affected [10, 11, 16-18].

Treatments for BMS patients have included Selective Serotonin Reuptake Inhibitors (SSRI), Serotonin-Norepinephrine Reuptake Inhibitors (SNRI), and multimodal antidepressants like vortioxetine (VO), which have shown efficacy in modulating pain perception and managing psychiatric comorbidities [13, 19, 20].

An open-label study by Adamo *et al*. revealed that VO significantly improved pain, depression, anxiety, and sleep quality, suggesting a new direction in BMS treatment [13]. These results were supported by a 12-month RCT comparing VO with other antidepressants, where VO demonstrated quicker antidepressant activity and pain control with fewer adverse events (AEs) [20].

Despite these advances, up to 60% of patients do not respond satisfactorily to monotherapy; a dose escalation and switching of antidepressants could be eventually recommended after an inadequate response [21, 22]. Pooled evidence from multiple trials of these drugs, as monotherapies, suggests only a partial benefit for most patients because of an incomplete efficacy and/or intolerable side effects at higher drug doses [23].

Evidence suggests that augmentation or combination strategies, such as adding voltage-gated calcium channel modulators like Pregabalin (PGB) to ongoing antidepressant pharmacotherapy, can improve treatment outcomes in patients with chronic pain and comorbid Major Depressive Disorder (MDD) not responding to standard therapies [23, 24].

Augmentation/combination strategies are frequently used in clinical routine care to improve treatment response in MDD and other chronic pain conditions such as fibromyalgia [25-27].

Polypharmacy is commonly used, and real-world prescribing studies indicate that most patients concurrently receive multiple treatments for chronic pain to address the limitations of monotherapy [28].

PGB is approved for the treatment of neuropathic pain and for generalized anxiety disorder (GAD), and it has also been considered in the treatment of BMS. The last chronic pain guidelines [29] strongly advocated for the augmentation of Ca^2+^-channel α2δ ligands such as PGB, in combination with duloxetine and tricyclic antidepressants, for pain relief in chronic pain treatment, recognizing the highly variable level of efficacy of pregabalin in enhancing the pain-relieving effects of specific antidepressants, particularly in neuropathic pain conditions [30].

Building upon these existing investigations, this study undertook a comparative trial involving a combination of PGB and VO *versus* PGB combined with another antidepressant (SSRI/SNRI) for BMS patients who showed inadequate response to antidepressant monotherapy after 12 weeks. The maximum dosage considered for PGB augmentation was 150 mg daily to avoid significant adverse events associated with the use of two drugs [31].

To our knowledge, this is the first trial specifically comparing VO, SSRI, and SNRI with PGB augmentation in the treatment of patients with BMS resistant to conventional monotherapy treatment.

This interventional, open-label, 52-week study aimed to compare the effectiveness and tolerability of VO and other antidepressants at fixed dosages with PGB augmentation in treatment-resistant BMS patients.

The primary objective of this study was to assess the effectiveness of VO and other antidepressants (SSRI and SNRI) at fixed dosages combined with PGB. Effectiveness was measured by the response rate, defined as the percentage of patients achieving a clinical response at 12, 24, 36, and 52 weeks.

### 1.1 Secondary Objectives Included

Acceptability: Determined by the percentage of participants discontinuing the trial for any reason, including the emergence of AEs, across each treatment group [32].

Tolerability: Assessed through the percentage of participants reporting specific AEs that impacted their quality of life for each treatment option [33].

The study also aimed to conduct a comparative analysis of these treatments regarding their response rate, safety, acceptability, and tolerability. This comparison was intended to elucidate the most effective therapeutic combination for BMS management. Furthermore, an analysis was performed to predict treatment responses, identifying patients unlikely to respond to the treatments and exploring potential reasons for such non-responsiveness.

## 2 MATERIALS AND METHODS

The recruitment has been performed in accordance with the ethical principles of the World Medical Association Declaration of Helsinki, approved by the University of Naples Federico II's Clinical Research Ethics Committee (Approval Number: 251/19) and registered under ClinicalTrials.gov (NCT06025474), was conducted in compliance with GDPR 2016/679 for data protection. Following CONSORT guidelines [34], the research spanned from January 1, 2023, to February 20, 2024, focusing on BMS treatment efficacy between Vortioxetine VO and an SSRI or SNRI supplemented with PGB.

The 450 BMS patients were initially randomized in a 2:1 ratio, resulting in 300 patients treated with VO (20 mg) and 150 patients treated with SSRIs/SNRIs in the following breakdown: 20 with P (20 mg), 20 with S (50 mg), 20 with C (20 mg), 20 with E (10 mg), and 70 with D (60 mg). The 2:1 randomization scheme was used to reflect real-world prescription practices in our Unit for the treatment of BMS.

Out of 450 assessed BMS patients, 203 non-responders to a 12-week monotherapy have been included in this 52-week, open-label, comparative clinical trial. For the unresponsive patients, there was no washout period between the initial 12-week treatment and the PGB augmentation. Group A included 136 patients treated with VO, and Group B comprised 67 patients treated with an SSRI or an SNRI. PGB 75 mg/day was added to the treatment at Time 0 for all 203 patients. At Time 1, 61 patients in Group A and 15 patients in Group B reached a complete clinical response after administration of 75 mg/day of PGB. Meanwhile, the unresponsive 75 patients in Group A and 52 patients in Group B received the additional 75 mg of PGB, reaching a total dosage of 150 mg. All antidepressants were administered at a fixed dose for 52 weeks; the dosage of antidepressants was chosen in line with the optimal dosage considered in the treatment of MDD in terms of efficacy, acceptability, and tolerability [35]. Fig. (**1**) shows the flow-chart of the study with a total of 203 individuals assessed for eligibility.

### 2.1 Eligibility Criteria for the Study

Eligibility for the study has been opened to individuals aged 18 to 85 years of all sexes, not based on gender, and does not include healthy volunteers. All the participants agreed to take part by providing their consent in writing.

### 2.2 Inclusion Criteria

- Patients must have a confirmed diagnosis of BMS as defined by the International Classification of Orofacial Pain, 1st edition (ICOP) [36].
- Complaints of oral burning must occur daily for more than 2 hours per day, persisting for over 3 months [36].
- Participants must have normal findings in blood tests, including but not limited to blood count, blood glucose levels, glycated haemoglobin, serum iron, ferritin, and transferrin levels.
- BMS patients who have previously been treated with one antidepressant for 12 weeks and have not responded to the treatment.

### 2.3 Exclusion Criteria

- Presence of any identifiable disease that could be recognized as a causative factor for BMS.
- A history of psychiatric disorders, neurological disorders, or organic brain diseases.
- A history of alcohol or substance abuse.
- Diagnosis of Obstructive Sleep Apnea Syndrome (OSAS).
- Uncontrolled hypertension, diabetes, HIV, narrow-angle glaucoma, or those participating in other investigational studies.
- Participants requiring continued treatment with medications known to adversely interact with study medications (*e.g*., quinolone antibiotics, warfarin, agents inhibiting serotonin reuptake) or those with hereditary problems of fructose intolerance, glucose-galactose malabsorption, or sucrose-isomaltase insufficiency.
- Pregnancy and lactation are also exclusion criteria. Women of childbearing potential must use a highly effective form of contraception throughout the study.

### 2.4 Arms and Interventions

The study includes multiple arms, each involving a combination of treatments to assess their efficacy in managing symptoms of a specific condition. The experimental arm consists of patients receiving VO (20 mg) and PGB (75 mg), administered orally once daily. For those in this group who do not respond adequately within 12 weeks while on the maximum dosage of VO, the dosage of PGB may be increased to 150 mg/day. VO and PGB serve as the primary drugs in this experimental setup, with 136 participants enrolled.

In addition to the experimental group, there are 5 comparator arms, each combining a different SSRI or SNRI with PGB at the starting dose of 75 mg/day. These include:

- Paroxetine (20 mg) for 10 participants.
- Sertraline (50 mg) for 8 participants.
- Citalopram (20 mg) for 8 participants.
- Escitalopram (10 mg) for 8 participants.
- Duloxetine (60 mg) for 33 participants.

This study employs a meticulous methodology to assess the efficacy and safety of VO and PGB compared to standard SSRIs/SNRIs and PGB in treating BMS resistant to initial therapy.

By administering all medications in encapsulated form once daily, with an option to increase PGB up to 150 mg for non-responders after 12 weeks, the trial ensures a structured and comprehensive evaluation.

This approach is further strengthened using multiple validated scales for efficacy assessment and rigorous monitoring of safety through adverse event reporting, aiming to provide in-depth insights into the comparative effectiveness and tolerability of these medication combinations.

The study employed a comprehensive set of primary and secondary outcome measures to evaluate the effectiveness of treatment BMS. Primary outcomes included the Visual Analog Scale (VAS) for pain intensity [37] and the Short-form McGill Pain Questionnaire (SF-MPQ) [37] for a multidimensional assessment of pain, both conducted at baseline and several intervals up to 52 weeks. Secondary outcomes encompassed the Hamilton Rating Scale for Depression (HAM-D) and the Hamilton Rating Scale for Anxiety (HAM-A) to assess psychiatric symptoms [38-40], and the Pittsburgh Sleep Quality Index (PSQI) for sleep quality [41], with evaluations paralleling those of the primary outcomes.

In addition, the Clinical Global Impression Scale (CGI) served as a comprehensive tool for evaluating overall patient progress, encompassing improvement, worsening, and the intensity of the condition [42]. At the outset (time 0), we used the CGI Severity of Illness (CGI-S) scale to establish a benchmark for disease severity. To gauge the overall positive change in patient condition following the initiation of treatment, we applied the CGI Improvement index (CGI-I). Moreover, the CGI Efficacy index (CGI-E), which offers a comparative analysis of therapeutic benefits against the severity of AEs based on the patient's condition at baseline, was administered at two critical junctures: the 24-week mark (time 2) and upon completion of a 52-week treatment period (time 4) [42].

All these scales were reviewed for completeness before collection and were administered in their Italian version by a single clinician to reduce inter-individual variability of judgment.

The efficacy of the treatment was evaluated based on the proportion of patients who achieved a clinical response. This determination of clinical response was aligned with the response criteria outlined in the MDD framework yet adapted specifically for chronic pain management according to Norbury and Seymour [43] and Amirdelfan *et al*. [44]. The criteria for a clinical response to the treatment were specified as follows:

- A decrease of the VAS and the SF-MPQ scores to levels between 1 and 2, indicating a substantial reduction in perceived pain.
- A reduction in the HAM-A and HAM-D scores by more than 50% or achieving scores of 7 or less on both scales, signifying a significant decrease in symptoms of anxiety and depression.

This structured approach provided a holistic evaluation of the treatments' effectiveness on pain, psychological well-being, and sleep quality in BMS patients [45].

The percentage of patients who reported the occurrence of specific AEs for each treatment (nausea, abdominal pain, dry mouth, dizziness, tremors, headache, blurred vision, difficulty concentrating, coordination problems, elevated serum, prolactin, somnolence, weight gain, increased appetite, constipation, sexual dysfunction, vivid dreams, peripheral edema, skin reactions, muscle pain), was evaluated and recorded at each control.

Electrocardiograms (ECG) were systematically performed at four-time points: baseline, 12 weeks, 24 weeks, and 52 weeks following the initiation of the study protocol. The primary focus of these ECG evaluations was to meticulously measure and analyze the QT and PR intervals [46].

The acceptability and tolerability of each treatment were measured as a percentage of patients who left the trial for any reason or due to the appearance of AEs before the end of the trial.

The study conducted a comprehensive comparison of the treatment modalities by examining response rates and assessing safety, acceptability, and tolerability. To further understand the dynamics of treatment efficacy, we performed an in-depth analysis of the sociodemographic and clinical factors influencing treatment outcomes. This evaluation aimed to distinguish between those who responded to the treatment and those who did not and to investigate the factors contributing to these differential responses. Through this analysis, we sought to identify key predictors of treatment success and to shed light on the reasons behind the varying levels of patient response.

### 2.5 Sample Size Determination

Utilizing an effect size of 1.86 derived from the HAM-D scale based on previous research by Adamo *et al*. [3], this study's sample size was calculated to ensure a power (1-Beta) of at least 99% and a significance level of no more than 1%. The calculation was facilitated by the GPower software (version 3.1.9), [47], ensuring robust statistical power for detecting treatment effects.

### 2.6 Statistical Methods

Statistical analyses were conducted using R software (version 4.1.2). Initial descriptive statistics provided an overview of the socio-demographic and clinical characteristics across the study groups, including means, standard deviations, medians, and inter-quartile ranges. To compare clinical outcomes between groups, either the Pearson Chi-Square or Fisher’s exact test was applied, depending on cell frequencies as recommended by Kim [48]. The Mann-Whitney U test facilitated comparisons of median values for outcomes such as VAS, SF-MPQ, HAM-D, HAM-A, and PSQI scores across multiple time points and CGI-I and CGI-E scores at specific intervals, with significance levels adjusted *via* the Bonferroni correction for multiple comparisons.

To refine our prediction model, we evaluated 115 variables covering a wide range of factors, from socio-demographic details to symptom patterns. A stepwise logistic regression, utilizing a forward selection method as outlined by Lee & Koval [49], identified a subset of predictors. This approach started with no variables and added them sequentially based on their contribution to model fit, continuing until no further variables significantly enhanced the model. Variables appearing in at least 10% of one group were considered to reduce variability and improve model accuracy.

After feature selection, two multivariate logistic regression models were developed to identify predictors of clinical response, incorporating variables such as socio-demographic factors, smoking, alcohol consumption, BMI, and physical activity. The odds ratio for each model was calculated. A sequential logistic regression analysis provided unadjusted coefficient estimates for predictors, followed by a comprehensive model analysis to determine adjusted coefficients, ensuring a thorough evaluation of potential predictors’ impact on clinical outcomes.

## 3 RESULTS

Table **1** analyses demographic variables, risk factors like smoking, alcohol use, body mass index (BMI), physical activity, menopause status, and disease onset duration in two groups: Group A received VO and PGB, while Group B was treated with an SSRI or an SNRI and PGB. No significant statistical differences were found in gender, level of education, family situation, risk factors, body mass index (BMI), physical activity, menopause status, and disease onset duration between the two groups.

The analysis of systemic diseases and drug consumption is shown in Table **2** and highlights a substantial prevalence of systemic diseases within the BMS patient population. Key findings include a high incidence of systemic conditions noted across both treatment groups, with 89% in Group A and 88.1% in Group B, demonstrating a significant presence of systemic diseases among BMS patients. The statistical analysis revealed no significant difference between the groups in terms of systemic disease prevalence (*p*-value: 0.818). Hypertension emerged as the most prevalent condition, affecting a majority in both groups, though no statistically significant differences were observed in the occurrence of hypertension, hypercholesterolemia, or previous myocardial infarction between the groups. This similarity extends to other conditions, such as hypercholesterolemia, gastroesophageal reflux disease (GERD), and hypothyroidism, with comparable proportions across both groups. Additionally, the study examined drug consumption patterns, noting that a significant portion of patients in both groups was on medications such as beta-blockers, ACE inhibitors, and angiotensin II receptor antagonists (ARBs), with similar patterns observed across the groups.

The prevalence of oral symptoms, the worst symptoms, the pattern of symptoms, and the location in both groups are presented in Table **3**. Burning, a hallmark symptom of BMS, was reported by 100% of patients in both groups.

No statistically significant differences were found in the symptoms, worst symptoms, patterns of symptoms, and location of burning reported by the two groups, except for subjective halitosis exclusively reported in Group B (10.4%; *p*-value: <0.001). Xerostomia (dry mouth), dysgeusia (altered taste), and globus pharyngeus (sensation of a lump in the throat) were the most frequent symptoms reported in association with burning. Xerostomia was reported by 68.4% of Group A and 61.2% of Group B, while dysgeusia was noted in 41.9% of Group A and 47.8% of Group B, globus pharyngeus was reported by 32.4% of Group A and 41.8% of the group B.

Table **4** provides a comprehensive overview of the changes in pain, psychiatric symptoms, and sleep quality over time.

A significant reduction in pain level was found for both treatment groups; at the end of the study, the median VAS scores had declined to 1; Group A demonstrated a more rapid decrease in pain scores at time 1 (Median and IQR: Group A: 6(5-7) and Group B 7(5-8); *p*-value: 0.006). Both groups reported similar improvements in pain perception as indicated by decreasing scores on the SF-MPQ, suggesting that both treatments are comparably effective in managing pain symptoms associated with BMS. Notable findings include significant improvements in anxiety and depression symptoms, especially pronounced in Group A from Time 0 to Time 1. While Group B also showed improvements, the early and marked progress in Group A (HAM-A and HAM-D; *p*-value 0.005) highlights the potential added benefit of their treatment regimen in addressing psychiatric symptoms.

The overarching conclusion drawn from Table **4** is that both treatment regimens offer effective symptom management for BMS, with a slight edge for Group A in terms of faster improvement in psychiatric symptoms. Additionally, the table underscores the methodological rigor in statistical analysis, incorporating the Pearson Chi-Square test, Fisher Exact Test, and Bonferroni correction for multiple comparisons, ensuring a robust interpretation of the significant changes observed across the clinical parameters measured at five distinct time points.

The time course of change from baseline in the VAS, SF-MPQ, HAM-A, HAM-D, and PSQI in group A and in group B is shown in Fig. (**2**).

Table **5** offers a detailed comparison of the clinical response and CGI scores over 52 weeks for the two groups.

The analysis reveals that both groups experienced improvements over the course of treatment. 84 (61.8%) in Group A and 39 (58.2.%) BMS patients in Group B showed a clinical response, respectively, after 52 weeks. The P-value for overall response was 0.761, indicating no significant difference between the groups in the proportion of responders.

However, Group A exhibited a stronger early response at Time 1 (Group A: 61; 44%; Group B: 15; 22.4% *p*-value: 0.002**).

Furthermore, by the end of the 52 weeks, despite no statistical differences found between Group A and Group B, Group A demonstrated slightly superior outcomes in terms of CGI-I and CGI-E, suggesting a more favourable treatment effect compared to Group B (Group A: CGI-I: 1 [1-2] and CGI-E: 1 [1-1]; Group B: CGI-I: 2 [1-2] and CGI-E: 1 [1-2]).

The time course of change from baseline scores on the CGI-I and CGI-E in group A and in group B is shown in Fig. (**3**).

Table **6** shows the prevalence, numbers, and type of AEs for both groups. The overall incidence of AEs was notably higher in Group B, with 20.8% of patients reporting AEs compared to 8.8% in Group A (*p*-value < 0.001). This difference was particularly significant in terms of patients experiencing more than one adverse event. This suggests that VO and PGB may offer a better tolerability profile compared with SSRI or SNRI and PGB.

Although AEs such as nausea, constipation, and dizziness were reported in both groups, their incidence did not differ significantly, indicating a similar tolerability for these side effects across treatments. However, Group B exhibited exclusive AEs, including dry mouth, QTc prolongation, elevated serum prolactin, and sexual dysfunction, delineating a distinctive adverse effect profile compared to Group A. This pattern may point to a heightened risk of specific AEs associated with the treatment regimen of Group B.

Furthermore, the occurrence of nausea and abdominal pain, which are typically linked with VO treatment, was relatively low in Group A (4 cases; 2.94%), and the frequency was not statistically different from that observed in Group B.

The administration of PGB at a maximum dosage of 150 mg demonstrated a notable safety profile, as it was not associated with several adverse effects commonly linked to its use. Specifically, at this dosage, patients did not report the occurrence of headaches, blurred vision, skin reactions, muscle pain, or PR prolongation.

Table **7** presents the results of a forward stepwise logistic regression analysis aimed at identifying predictors of clinical response in a treatment study. Smoking, physical activity, and medication use such as Angiotensin II Receptor Blockers (ARBs) and paroxetine and QTC value were predictors of a positive clinical response.

The beta coefficient for smokers is 1.03 with an odds ratio (OR) of 2.79, indicating that smokers are approximately 2.79 times more likely to achieve a clinical response compared to non-smokers, with a strongly significant *p*-value of 0.006.

Individuals engaging in physical activity have a beta coefficient of 1.51, an OR of 4.51, and a strongly significant p-value of 0.007.

The use of ARBs is associated with a beta of 1.02 and an OR of 2.79, showing that individuals on these medications are nearly three times more likely to have a positive clinical response, with a p-value of 0.015 indicating moderate significance. This could imply a potential pharmacological synergy or an independent effect of ARBs on the condition being treated. The beta coefficient for the use of Paroxetine is 1.66, with an OR of 5.24 and a moderately significant p-value of 0.046. This finding indicates that patients treated with Paroxetine are over five times more likely to experience a clinical response.

Every unit increase in QTc value is associated with a slight increase in the odds of a clinical response (OR=1.02) with a beta of 0.02. The *p*-value of 0.016 suggests moderate significance, indicating a potential but subtle effect of cardiac electrical activity on treatment response.

Table **8** summarizes the results of multivariate logistic regression analyses predicting clinical response in two treatment groups. The analyses explore various predictors across two models for each group, providing insights into factors influencing treatment outcomes.

Smoking and physical activity emerge as significant predictors of clinical response in both models for Group A, with smokers showing an increased likelihood of a positive response (OR = 2.71 in Model 1 and OR = 2.80 in Model 2, *p <* 0.05) and physical activity associated with a higher chance of a positive response (OR = 2.99 in Model 1, *p =* 0.080; OR = 4.02 in Model 2, *p =* 0.038). The introduction of ARBs and QTc value in Model 2 for Group A significantly predicts clinical response, with ARBs having an OR of 3.62 (*p =* 0.021) and QTc value an OR of 1.02 (*p =* 0.037), indicating their positive influence on treatment efficacy. The R2 change from Model 1 to Model 2 in Group A (6.1%, *p =* 0.004) signifies a meaningful improvement in model fit, suggesting that the additional predictors in Model 2 contribute significantly to explaining the variance in clinical response.

In Group B, model 1 and model 2 show that most predictors do not reach statistical significance, indicating a more nuanced relationship between these variables and clinical response in Group B.

However, in Model 2, Paroxetine stands out with an OR of 9.75 (*p =* 0.019), suggesting a strong association with a positive clinical response. This might indicate a specific effectiveness of Paroxetine in combination with PGB in this group. The R2 change in Group B from Model 1 to Model 2 (9.2%, *p =* 0.038) indicates a substantial improvement in explaining the variance in clinical response, emphasizing the role of paroxetine and possibly other introduced variables.

## 4 DISCUSSION

BMS continues to challenge clinicians in selecting appropriate treatments to manage its complex symptomatology effectively. This study builds on the premise that combining antidepressants with PGB — a strategy proven effective in other chronic pain conditions — may offer a promising avenue for BMS management [24, 30, 50, 51]. The findings from this prospective trial suggest that both VO and an SSRI or SNRI when augmented with PGB, provide significant symptom improvement in treatment-resistant BMS.

The use of antidepressants in managing BMS is supported by a wealth of research, including recent systematic reviews [1, 52] that found these medications to be effective in both short-term and long-term evaluations. This suggests that antidepressants can play a crucial role in alleviating the symptoms associated with BMS.

Specifically, VO offers a novel approach for its unique, multimodal action not only inhibits serotonin reuptake but also modulates various serotonin receptors [53, 54]. This dual functionality is significant for enhancing neuroplasticity and improving the regulation of mood and cognitive functions—areas often impacted by chronic pain conditions [12, 55, 56]. Consequently, VO's comprehensive mechanism presents a promising avenue for treating chronic pain, including BMS, by addressing both the physical and psychological aspects of these conditions. The pain-relieving properties of VO have been confirmed both experimentally and clinically.

The study by Zuena *et al*. [57] demonstrated VO's analgesic action in a mouse model of chronic neuropathic pain, comparing its efficacy to venlafaxine and fluoxetine. VO caused robust analgesia comparable to venlafaxine, unlike fluoxetine, and did not alter motor activity, suggesting its effectiveness in neuropathic pain treatment, particularly for patients with comorbid depression and cognitive dysfunction [57].

Subsequently, research by Todorović *et al*. revealed that VO can significantly lessen pain behavior and inflammation-related hyperalgesia in a dose-dependent manner [58]. The analgesic effect was mediated through multiple receptors, including 5‐HT1B/1D serotonergic, α2/β1‐adrenergic, muscarinic, and nicotinic cholinergic, CB1/CB2 cannabinoid, and adenosine A1 receptors, suggesting VO’s potential as a versatile treatment for inflammatory pain [58, 59].

Yücel *et al*. further investigated VO’s pain-relief capabilities, showing significant increases in response latency to painful stimuli in mice. This effect, mediated by serotonergic, adrenergic, and opioid receptors, highlights the importance of these neurotransmissions in VO’s analgesic action [59].

Clinical evidence further supports the effectiveness of VO. Adamo and colleagues, in an open-label flexible-dose pilot study involving 30 BMS patients, reported that VO significantly improved pain, depression, anxiety, and sleep quality scores [60] also compared with four of the most prescribed antidepressants for BMS management [20]. These studies emphasize VO's capability not only to alleviate physical symptoms of BMS but also to address the psychological aspects intertwined with this condition [13].

Moreover, SSRIs and SNRIs work by increasing the levels of serotonin and/or norepinephrine in the brain, effectively modulating pain pathways as well as mood [61, 62]. These medications have shown diverse effectiveness in treating neuropathic pain conditions like fibromyalgia and BMS [61, 63]. This suggests their promising role in managing BMS, either as standalone treatments or in synergy with other medications, such as PGB, for improved therapeutic outcomes.

PGB, on the other hand, is a gabapentinoid that binds to the alpha-2-delta subunit of voltage-gated calcium channels in the central nervous system [64]. This binding reduces calcium influx at nerve terminals, leading to a decreased release of several neurotransmitters, including glutamate, noradrenaline, and substance P, which are involved in the transmission of pain signals and epileptiform activity [65]. Therefore, PGB modulates neuronal activity, which can lead to reductions in pain, improvements in sleep quality, and the alleviation of mood disorders and the associated anxiety [66, 67].

The therapeutic potential of PGB for chronic pain and BMS has been underscored by various studies. For instance, Ito *et al*. [68] highlighted PGB's effectiveness in treating BMS in five patients who did not respond to SNRI treatments, positioning PGB as a viable option for BMS management. This finding is supported by Choi *et al*. [6], who reported significant benefits of PGB in a study involving 33 BMS patients, with dosages ranging from 75 to 150 mg daily.

The current study is pioneering in evaluating the VO and PGB combination for treating a chronic pain condition like BMS, building on previous research that has already investigated and supported the efficacy of combining SSRI/SNRI with PGB in patients with MDD and chronic pain [69].

Notably, a landmark randomized, placebo-controlled trial by Arnold *et al*. [70] investigated the efficacy and safety of PGB in fibromyalgia patients who were also receiving SSRI or SNRI treatment for comorbid depression. The findings were significant, revealing that PGB led to substantial reductions in pain and notable improvements in anxiety, depression, patient functioning, and sleep quality when compared to a placebo [70]. These findings highlight the potential of using PGB as an additional treatment for managing fibromyalgia pain in patients also receiving SSRIs or SNRIs for depression. Furthermore, it suggests that combining VO and PGB could improve treatment results for chronic pain conditions like BMS, providing new insights and optimism for patients dealing with this difficult condition.

In this study, analyzing baseline characteristics such as socio-demographics (gender, age, education, family), oral symptoms, and systemic diseases alongside drug use showed no significant differences between groups. This uniformity supports attributing treatment efficacy, side effects, and patient satisfaction solely to the treatment methods under study. The high prevalence of systemic diseases and drug use in both groups emphasizes considering the overall health of BMS patients when selecting treatment to avoid exacerbating these conditions.

Both treatments led to significant improvements in pain perception over time, as indicated by reductions in VAS and SF-MPQ scores in both groups. Additionally, significant decreases in HAM-A, HAM-D, and PSQI scores over time suggest the effectiveness of both treatments in managing anxiety, depressive symptoms, and sleep disturbances. The combination of the two drugs further enhanced treatment response, with 62% of patients in Group A and 58% in Group B showing a complete clinical response after 52 weeks. Notably, 44.8% of Group A patients achieved clinical response at time 1 without requiring an increase in PGB dosage over 75 mg, compared to only 22.3% in Group B. Despite no dosage adjustments after time 2, BMS patients continued to improve until reaching clinical response, supporting the notion of prolonged treatment recommended for this disease.

Although both pharmacological strategies demonstrated their efficacy in managing patients affected by BMS, a notable quick and effective response was observed from the VO and PGB regimen. This early response may suggest a potential advantage of the VO and PGB combination in providing quicker relief from symptoms, an aspect that could be pivotal for patients seeking immediate symptom management. These findings corroborate prior research indicating that VO demonstrates more rapid antidepressant effects and pain management compared to SSRIs and SNRIs [20]. However, future studies should further elucidate the long-term efficacy of the VO and PGB combination, particularly in comparison to SSRIs and SNRIs.

In addition, the analysis of AEs revealed a higher incidence in Group B (SSRI/SNRI and PGB: 20.8%), suggesting a better tolerability profile for the VO and PGB combination (Group A: 8.8%). This aspect of treatment selection is particularly relevant in the clinical management of BMS, where the chronic nature of the syndrome requires long-term treatment strategies that not only are effective but also minimize patient discomfort and potential side effects.

This finding may also be explained because most patients of group B (52; 77.7%) were treated with a higher dosage of PGB compared with group A because the BMS patient in this group didn’t achieve clinical response at time 1.

However, the administration of PGB at a maximum dosage of 150 mg daily exhibited a favorable safety profile because AEs commonly associated with its use, including headaches, blurred vision, skin reactions, muscle pain, and PR prolongation, were notably absent in the reported data. This absence of typical AEs suggests that the controlled dosage likely played a pivotal role in minimizing these undesirable effects, underscoring the importance of dosage management in the tolerability of PGB.

Although no studies on the combination of VO and PGB currently exist in the literature, the VIVRE study by McIntyre *et al*. [71], which directly compares VO and desvenlafaxine, reveals a numerical advantage of VO in achieving symptomatic and functional remission among patients with partial responses to initial SSRI therapy. This outcome hints at the potential superiority of VO as a standalone treatment in certain patient demographics [71].

Moreover, the overall acceptability of both treatment regimens was high, as evidenced by the low discontinuation rates among participants. Specifically, in Group A, only 6 individuals (representing 4.4% of the group) discontinued the trial, while Group B saw a slightly higher discontinuation rate, with 6 individuals (8.9%) opting out. These low discontinuation rates further indicate a positive reception to the treatments, with the lower rate in Group A suggesting a marginally better acceptability compared to Group B. This could potentially be attributed to the effective management of side effects, including the strategic limitation of PGB dosage, which likely contributed to enhancing patient adherence and overall treatment satisfaction.

The faster onset of action and the better tolerability profile with lower AEs associated with the VO-PGB combination suggest a potentially more favorable treatment option that could significantly impact patient management strategies.

Nevertheless, the landscape of psychiatric treatment is complex and marked by divergent findings. A notable example is the recent VESPA study conducted by Ostuzzi *et al*. [72], which explored the efficacy and tolerability of VO in a cohort of 357 older adults with MDD. Contrary to expectations, this study revealed that VO did not exhibit a superior tolerability profile or an improved response rate to SSRIs. A critical analysis of the study’s demographics revealed notable differences, particularly concerning the prevalence of systemic diseases. While in this study, a high incidence of systemic comorbidities among participants (89% in the VO group and 88.1% in the SSRI/SNRI group) has been reported, the VESPA study's cohort did not mirror this trend, with lower percentages of patients with systemic diseases in both the VO (43%) and SSRI groups (43.8%).

This variance brings to light critical considerations regarding the relationship between chronic pain, concurrent health conditions, and the effectiveness of psychiatric medications. It hints at the possibility that patients grappling with chronic pain conditions, such as BMS, who typically present with a complex array of multiple comorbidities and are often subjected to polypharmacy, might exhibit varied responses to psychiatric treatments compared to those solely diagnosed with MDD. This insight opens the discussion on the intricate dynamics of patient health in BMS and its potential impact on treatment outcomes, including response rates and tolerability to medications.

Within this nuanced framework, the VO-PGB treatment strategy emerges as a potentially viable option, especially for BMS patients who have shown partial or no response to initial antidepressant monotherapy. Its favorable safety profile renders this combination therapy an appealing choice, particularly for the elderly or those patients managing multiple systemic comorbidities [73, 74]. This demographic is especially vulnerable to the challenges of polypharmacy, including a higher risk of drug-drug interactions and an increased sensitivity to adverse effects. Therefore, a treatment option that minimizes AEs while providing effective symptom relief is highly desirable in BMS patients.

Incorporating the VO-PGB combination as a second-line treatment strategy for BMS could thus offer a tailored approach, enhancing the quality of care for patients who necessitate a balance between efficacy and safety due to their complex medical backgrounds. This consideration is crucial for optimizing therapeutic outcomes and improving overall patient well-being, particularly in populations that are more vulnerable to the AEs of conventional treatments.

The results of the study have highlighted the significance of lifestyle factors, specific medications, and physiological measures in predicting clinical response. Notably, the positive association of smoking with clinical response is unusual and warrants further investigation to understand the underlying mechanisms. The strong predictive value of physical activity underscores the importance of lifestyle modifications as part of treatment plans. The associations with ARBs and Paroxetine suggest that certain medications may enhance treatment effectiveness, possibly through mechanisms specific to the condition being treated or through general health improvements [75]. Physical activity is known to exert multifaceted effects on neurobiology, including modulation of neurotransmitter levels, promotion of neuroplasticity, and enhancement of overall brain health. Consequently, its integration into treatment strategies may augment therapeutic outcomes in psychiatric disorders. Similarly, ARBs and Paroxetine possess distinct pharmacological profiles within their respective classes, potentially offering unique mechanisms of action that contribute to treatment response. Expounding upon these pharmacodynamic nuances could shed light on why these medications were specifically chosen for investigation. Moreover, the observation of an increase in QTc values prompts consideration of its implications on treatment efficacy. While QTc prolongation may reflect underlying cardiac concerns that could impact medication metabolism, it may also directly influence neurotransmitter systems relevant to psychiatric symptomatology. However, the relationship between QTc prolongation and treatment response is complex. SSRIs and pregabalin can prolong the QTc interval, which may reflect an underlying autonomic imbalance rather than increased drug efficacy.

As Nagamine and Watanabe [76] suggest, the QTc interval may indicate autonomic imbalance, with a shorter interval and high pain catastrophizing reflecting increased sympathetic tone. This heightened tone could impact neurotransmitter systems and psychiatric symptoms through greater autonomic activation. Thus, our study underscores the importance of comprehensively evaluating various biological and pharmacological factors to better predict and optimize treatment response in psychiatric populations. Our research shows that smoking is linked to increased responses to certain antidepressants, likely due to nicotine's effects on the brain. Nicotine boosts dopamine levels, making antidepressants more effective and improving mood faster in smokers than non-smokers [77]. However, smoking can also reduce the effectiveness of antidepressants by speeding up their breakdown in the body. Similarly, smoking affects pain perception and treatment in complex ways [78]. Nicotine may enhance the effects of natural pain relievers and reduce inflammation-related pain, but smokers may develop a tolerance to pain medications and experience variable pain relief [79]. Considering the serious health risks of smoking, it is not a recommended treatment. Future research should explore safer ways to harness nicotine's benefits for pain and depression without the harmful effects of smoking.

The limited explanatory power of the current models highlights the intricate nature of BMS treatment outcomes, which are influenced by a multifaceted web of factors, including lifestyle choices, the use of specific medications, and various clinical parameters. It suggests that there are additional, unaccounted-for elements that significantly impact the effectiveness of therapeutic interventions. This complexity points to the potential for genetic, environmental, psychological, and other physiological factors to play critical roles in how individuals respond to treatment [80].

Given this complexity, future research should aim to uncover these hidden variables and better understand their interconnections. Doing so could lead to the development of more comprehensive and personalized treatment plans tailored to the unique circumstances of each patient.

## 5 LIMITATIONS

The open-label nature of the study, while facilitating real-world applicability, introduces potential biases that may affect the objectivity of the findings. Furthermore, the challenges of grouping SSRIs and SNRIs together highlight potential limitations in interpretation. Specifically, the variability in side effects within this pharmacodynamically heterogeneous class of medications underscores the nuanced nature of assessing the tolerability and acceptability of SSRIs and SNRIs as a group. Future studies should aim to incorporate double-blind methodologies and explore biomarkers or other objective measures to enhance predictive capabilities.

## CONCLUSION

This study significantly enriches the existing knowledge on the management of BMS, underscoring the necessity for a tailored, patient-focused approach in pharmacological treatment. It emphasizes the importance of not only evaluating the effectiveness and tolerability of treatment options but also considering the comprehensive context of an individual's health and lifestyle.

The augmentation of PGB to VO or an SSRI or SNRI emerges as a promising treatment avenue for BMS, particularly for those patients who have not found relief through monotherapy. The VO-PGB combination, with its rapid onset and improved tolerability profile, could represent a potentially superior treatment option. This could be especially beneficial for specific patient groups, including the elderly or those with multiple systemic comorbidities, who are at a higher risk for adverse effects due to polypharmacy.

These insights advocate for a nuanced approach in selecting BMS treatment strategies, where both the efficacy and safety profiles are balanced, aiming to optimize symptom relief while minimizing the risk of side effects in a perspective of personalized care. This study sheds light on the intricate factors—demographic, lifestyle, and clinical—that influence treatment outcomes, highlighting the value of a holistic patient assessment in devising effective therapeutic plans.

However, the path to refining BMS treatment strategies remains ongoing. Future research should delve into understanding the underlying mechanisms that dictate treatment responses, investigating the impact of genetic, psychological, and lifestyle elements, and developing predictive models for more precise treatment guidance to promote personalized, efficacious, and patient-centered pathways of care.

## Figures and Tables

**Fig. (1):**
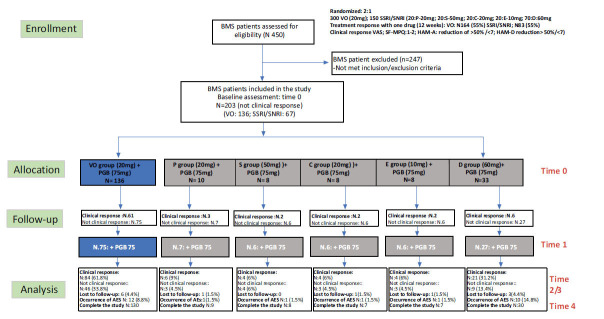
Flow-chart of the study. **Abbreviations:** AES: Adverse Events; BMS: Burning Mouth syndrome; C: Citalopram; D: Duloxetine; E: Escitalopram; HAM-A: Hamilton Anxiety; HAM-D: Hamilton depression; P: Paroxetine; PGB: Pregabalin; S: Sertraline; SF-MPQ: Short form of Mc Gill Pain Questionnaire; SSRI: Selective Serotonin Reuptake Inhibitor; SNRI: Dual Serotonin and Norepinephrine Reuptake Inhibitor; V: Vortioxetine: VAS: Visual Analogue Scale.

**Fig. (2):**
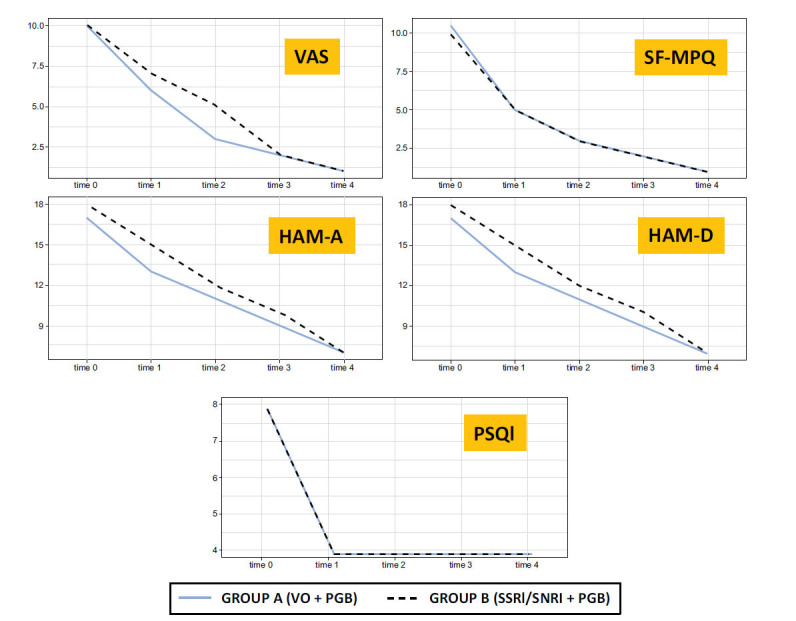
Time course of change from baseline in the VAS, SF-MPQ, HAM-A, HAM-D, and PSQI in group A and in group B. **Abbreviations:** HAM-A Hamilton Anxiety; HAM-D Hamilton Depression; PSQI, Pittsburgh Sleep Quality Index; SF-MPQ: Short form of McGill Pain Questionnaire; PGB: Pregabalin; SSRI: Selective Serotonin Reuptake Inhibitor; SNRI: Dual Serotonin and Norepinephrine Reuptake Inhibitor VAS: Visual Analogue Scale; VO: Vortioxetine.

**Fig. (3):**
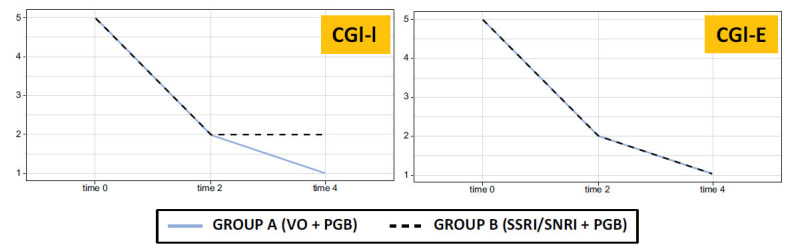
Time course of change from baseline scores on the CGI-I and CGI-E in group A and in group B. **Abbreviations:** CGI-E: Clinical Global Impression Efficacy, CGI-I- Clinical Global Impression-Improvement; PGB: Pregabalin; SSRI: Selective Serotonin Reuptake Inhibitor; SNRI: Dual Serotonin and Norepinephrine Reuptake Inhibitor VAS: Visual Analogue Scale; VO: Vortioxetine.

**Table 1:** Socio-demographic profile, risk factors, and disease onset in 203 BMS patients: 136 treated with vortioxetine and pregabalin (group A), and 67 treated with an SSRI or SNRI and pregabalin (group B).

**Demographic Variables**	**GROUP A (VO + PGB)** 136 (67.3%)	**GROUP B (SSRI/SNRI + PGB)** 67 (32.7)	***P*-value**
**Gender** Male Female	**Frequency (%)** 35 (25.7) 101 (74.3)	**Frequency (%)** 16 (23.9) 51 (76.1)	0.909
**Age** (in years)	**Mean ± SD** 64.7 ± 12.4	**Mean ± SD 66.2** ± 14	0.458
**Education** (in years)	**Mean ± SD** 9.46 ± 4.44	**Mean ± SD 8.94** ± 4.66	0.453
**Family situation** • Single • Married • Divorced • Widowed	**Frequency (%)** 8 (5.9) 99 (72.8) 7 (5.1) 22 (16.2)	**Frequency (%)** 4 (6) 52 (77.6) 1 (1.5) 10 (14.9)	0.713
**Employment** • Employed • Unemployed • Retired	**Frequency (%)** 34 (25) 56 (41.2) 48 (35.3)	**Frequency (%)** 19 (28.4) 28 (41.8) 20 (29.9)	0.664
**Risk factors**	**Frequency (%)**	**Frequency (%)**	***P*-value**
**Smoking** • Never • <5 cigarettes • 5-10 cigarettes • 10-15 cigarettes • >15 cigarettes	100 (73.5) 5 (3.7) 5 (3.7) 14 (10.3) 12 (8.8)	49 (73.1) 6 (9) 4 (6) 3 (4.5) 5 (7.5)	0.302
**Alcohol use** • Never • Yes (1 unit) • Yes (2 units) • Yes (>2)	115 (84.6) 15 (11) 5 (3.7) 1 (0.7)	56 (83.6) 8 (11.9) 2 (3) 1 (1.5)	0.951
**Body Mass Index (kg/m2)** • BMI<18.5 • BMI: 18.5-24.9 *normal* • BMI: 25.0-29.9 *overweight* • BMI: 30-34 *class I obesity* • BMI: 35-39.99 *class II obesity* • BMI>40 *class III obesity* **BMI**	0 (0) 49 (36) 68 (50) 17 (12.5) 1 (0.7) 1 (0.7) **MEAN ± SD** 26.4 ± 3.47	0 (0) 15 (22.4) 44 (65.7) 6 (9) 1 (1.5) 1 (1.5) **MEAN ± SD** 26.9 ± 3.79	0.366
**Physical Activity (Yes)**	19 (14)	4 (6)	0.146
**Menopause (Yes)**	93 (68.4)	41 (61.2)	0.390
**Disease Onset**	**Mean ± SD** 24 ± 38	**Mean ± SD** 29.6 ± 33.7	0.283

**Note**: The significance difference between means was measured by the t-student test. The significance difference between percentages was measured by the Pearson Chi Square test. **Abbreviations**: BMS: Burning Mouth Syndrome; BMI: Body Mass Index; PGB: Pregabalin; SSRI: Selective Serotonin Reuptake Inhibitor; SNRI: Dual Serotonin and Norepinephrine Reuptake Inhibitor; VO: Vortioxetine.

**Table 2:** Prevalence of systemic diseases and drug consumption in 203 BMS patients: 136 treated with Vortioxetine and Pregabalin (group A), and 67 treated with an SSRI or SNRIs and pregabalin (group B).

**Systemic Diseases**	**GROUP A (VO + PGB) Frequency (%) 136 (67.3%)**	**GROUP B (SSRI/SNRI + PGB) Frequency (%)** 67 (32.7)	***P*-value**
Yes Not	121(89) 15 (11)	59 (88.1) 8 (11.9)	0.818
Hypertension	77 (56.6)	34 (50.7)	0.456
Hypercholesterolemia	53 (39)	24 (35.8)	0.759
Previous myocardial infarction	4 (2.9)	3 (4.5)	0.687
Hypothyroidism	25 (18.4)	10 (14.9)	0.693
Hyperthyroidism	0 (0)	1 (1.5)	0.330
Endocrine Disease	1 (0.7)	0 (0)	1.000
Gastroesophageal reflux disease	26 (19.1)	13 (19.4)	1.000
Neoplastic diseases	4 (2.9)	4 (6)	0.443
Asthma	5 (3.7)	2 (3)	1.000
HBV infection	3 (2.2)	3 (4.5)	0.399
HCV infection	1 (0.7)	2 (3)	0.254
Neurological disorders	2 (1.5)	2 (3)	0.600
Others	1 (0.7)	2 (3)	0.254
**Drug Consumption**	**Frequency (%)**	**Frequency (%)**	***P*-value**
Yes Not	106 (77.9) 30 (22.1)	50 (74.6) 17(25.4)	0.600
Beta-blockers	24 (17.6)	13 (19.4)	0.847
ACE-inhibitors	15 (11)	12 (17.9)	0.191
Angiotensin II receptor antagonists (ARBs)	28 (20.6)	11 (16.4)	0.571
Thiazide Diuretics	21 (15.4)	7 (10.4	0.392
Calcium Channel blockers	12 (8.8)	7 (10.4	0.799
Antiplatelets	34 (25)	25 (37.3)	0.073
Blood thinner	11 (8.1)	4 (6)	0.777
Statins	32 (23.5)	14 (20.9)	0.724
Ezetimibe	4 (2.9)	0 (0)	0.305
Proton pump inhibitors	35 (25.7)	13 (19.4)	0.381
Levothyroxine sodium	16 (11.8)	9 (13.4)	0.821
Bisphosphonates	2 (1.5)	4 (6)	0.094
Others	5 (3.7)	5 (7.5)	0.303

**Note**: A significance difference between the percentages was measured by the Pearson Chi-Square test. When one or more cells contain a frequency less than 5 then the Fisher Exact Test was used. Significant with Bonferroni correction should be 0.003. **Abbreviations**: BMS: Burning Mouth Syndrome; PGB: Pregabalin; SSRI: Selective Serotonin Reuptake Inhibitor; SNRI: Dual Serotonin and Norepinephrine Reuptake Inhibitor; VO: Vortioxetine.

**Table 3:** Frequency of Oral Symptoms, pattern of symptoms, and location in 203 BMS patients: 136 were treated with vortioxetine and pregabalin (group A), and 67 were treated with an SSRI or SNRI and pregabalin (group B).

**Oral Symptoms**	**GROUP A (VO + PGB) Frequency (%)** 136 (67.3%)	**GROUP B (SSRI/SNRI + PGB) Frequency (%)** 67 (32.7)	***P*-Value**
Burning	136 (100)	67 (100)	1.000
Xerostomia	93 (68.4)	41 (61.2)	0.346
Dysgeusia	57 (41.9)	32 (47.8)	0.455
Globus Pharingeus	44 (32.4)	28 (41.8)	0.213
Subjective change in tongue morphology and color	31 (22.8)	5 (7.5)	0.006
Sialorrhea	27 (19.9)	8 (11.9)	0.174
Intraoral foreign body sensation	20 (14.7)	15 (22.4)	0.235
Tingling sensation	23 (16.9)	4 (6)	0.046
Itching	13 (9.6)	9 (13.4)	0.473
Oral dyskinesia	6 (4.4)	5 (7.5)	0.510
Occlusal Dysesthesia	5 (3.7)	5 (7.5)	0.303
Dysosmia	5 (3.7)	3 (4.5)	0.721
Subjective Halitosis	0 (0)	7 (10.4)	<0.001**
**Worst Symptom**	**Frequency (%)**	**Frequency (%)**	***P*-Value**
Burning	110 (80.9)	64 (95.5)	0.005
Xerostomia	9 (6.6)	0 (0)	0.031
Change in tongue morphology/color	5 (4.8)	0 (0)	0.173
Dysgeusia	2 (1.5)	0 (0)	1.000
Globus	2 (1.5)	0 (0)	1.000
Sialorrhea	3 (2.2)	0 (0)	0.552
Intraoral foreign body sensation	2 (1.5)	1 (1.5)	1.000
Occlusal Dysesthesia	2 (1.5)	1 (1.5)	1.000
Oral Dyskinesia	1 (0.7)	1 (1.5)	0.552
**Pattern of Symptoms**	**Frequency (%)**	**Frequency (%)**	***P*-Value**
Same in the morning/afternoon/evening Worse in the afternoon/evening Continuous Intermittent Improving during meal (yes)	73 (53.7) 49(36) 79(58.1) 53(39) 29(21.3)	34 (50.7) 31(46.3) 44(65.7) 22(32.8) 17(25.4)	0.773 0.136 0.299 0.395 0.517
**Location of Pain/Burning**	**Frequency (%)**	**Frequency (%)**	***P*-Value**
Tongue	130 (95.6)	65 (97)	1.000
Palate	90 (66.2)	46 (68.7)	0.753
Lips	82 (60.3)	42 (62.7)	0.762
Gums	84 (61.8)	43 (64.2)	0.760
Cheeks	83 (61)	39 (58.2)	0.761
Floor of the Mouth	74 (54.4)	38 (56.7)	0.767
Trigone	73 (53.7)	38 (56.7)	0.765

**Note**: A significant difference between the percentages was measured by the Pearson Chi Square test. When one or more cells contain a frequency less than 5 then the Fisher Exact Test was used. **Significant with Bonferroni correction 0.002 for the oral symptoms, pattern of symptoms and worst symptom. **Significant with Bonferroni correction 0.006 for the location of pain/burning. **Abbreviations**: BMS: Burning Mouth Syndrome; PGB: Pregabalin; SSRI: Selective Serotonin Reuptake Inhibitor; SNRI: Dual Serotonin and Norepinephrine Reuptake Inhibitor; VO: Vortioxetine.

**Table 4:** Pain, Psychological profile, and sleep evaluation in 203 BMS patients: 136 were treated with vortioxetine and pregabalin (group A), and 67 were treated with SSRIs or SNRIs and pregabalin (group B).

**Clinical Parameters**		**Time 0**		**Time 1**		**Time 2**		**Time 3**		**Time 4**	
		**Median; [IQR]**	***P*-value**	**Median; [IQR]**	***P*-value**	**Median; [IQR]**	***P*-value**	**Median; [IQR]**	***P*-value**	**Median; [IQR]**	***P*-value**
VAS	GROUP B SSRI/SNRI + PGB	10 [10-10]	0.292	6 [5-7]	0.006**	3 [3-5]	0.024	2 [1-3]	0.090	1 [1-1]	0.546
				7 [5-8]		5 [3-5]		2 [2-3]		1 [1-1]	
SF-MPQ	GROUP A VO + PGB	10.5 [7-13]	0.912	5 [3-8]	0.975	3 [2-5]	0.491	2 [1-3]	0.860	1 [1-2]	0.862
	GROUP B SSRI/SNRI + PGB	10 [7-12]		5 [4-7]		3 [2-5]		2 [1-3]		1 [1-2]	
HAM-A	GROUP A VO + PGB	17 [15-20]	0.067	13 [11-15]	0.005**	11 [9-12]	0.061	9 [7-10]	0.146	7 [7-9]	0.820
	GROUP B SSRI/SNRI + PGB	18 [15-22]		15 [12-18]		12 [10-15]		10 [7-11]		7 [7-10]	
HAM-D	GROUP A VO + PGB	17 [14-20]	0.095	13 [11-15]	0.005**	11 [9-12]	0.015	9 [7-10]	0.139	7 [7-9]	0.639
	GROUP B SSRI/SNRI + PGB	18 [15-22]		15 [12-18]		12 [10-15]		10 [7-11]		7 [7-10]	
PSQI	GROUP A VO + PGB	8 [7-9]	0.253	4 [4-4]	0.387	4 [4-4]	0.260	4 [4-4]	0.900	4 [4-4]	0.489
	GROUP B SSRI/SNRI + PGB	8 [8-10]		4 [4-4]		4 [4-4]		4 [4-4]		4 [4-4]	

**Note**: IQR is the interquartile range. The significance of differences between medians was measured by performing the Mann-Whitney test. **Significant with Bonferroni correction 0.01. **Abbreviations:** BMS, burning mouth syndrome; ESS, Epworth Sleepiness Scale; HAM-A Hamilton Anxiety; HAM-D Hamilton Depression; PGB: Pregabalin; PSQI, Pittsburgh Sleep Quality Index; SF-MPQ: Short form of McGill Pain Questionnaire; SSRI: Selective Serotonin Reuptake Inhibitor; SNRI: Dual Serotonin and Norepinephrine Reuptake Inhibitor VAS: Visual Analogue Scale; VO: Vortioxetine.

**Table 5:** Clinical Global Impression Severity (CGI-S), Clinical Global Impression Improvement (CGI-I), and Clinical Global Impression Efficacy (CGI-E) variation in 203 BMS patients: 136 treated with vortioxetine and pregabalin (group A) and 67 treated with SSRIs or SNRIs and pregabalin (group B).

**Clinical Response**	**GROUP A VO + PGB Frequency (%)** 136 (67.3%)	**GROUP B SSRI/SNRI + PGB Frequency (%)** 67 (32.7)	***P*-value**
**After 52 weeks (yes) Time 1 (yes) Time 2 Time 3 Time 4**	**84 (61.8) 61(44.8) 10 (7.4) 10 (7.4) 3(2.2)**	**39 (58.2) 15 (22.4) 10 (14.9) 10 (14.9) 4 (6)**	0.761 0.002** 0.131 0.131 0.222
**CGI-Severity (CGI-S)**	**GROUP A VO + PGB Median [IQR]**	**GROUP B SSRI/SNRI + PGB Median [IQR]**	***P*-value**
**Time 0**	5 [4-5]	5 [4-5]	0.238
**CGI-Improvement (CGI-I)**	-	-	-
**Time 2: 24 weeks**	2 [1-2]	2 [2-3]	0.238
**Time 4: 52 weeks**	1 [1-2]	2 [1-2]	0.046
**Clinical Global Impression Efficacy (CGI-E)**	-	-	-
**Time 2: 24 weeks**	2 [1-5]	2 [1-2.5]	0.596
**Time 4: 52 weeks**	1 [1-1]	1 [1-2]	0.101

**Note**: A significance difference between the percentages was measured by the Pearson Chi Square test. When one or more cells contain a frequency less than 5 then the Fisher Exact Test was used. IQR is the interquartile range. The significance of differences between medians was measured by performing the Mann-Whitney test. **Significant with Bonferroni correction 0.01. **Abbreviations:** BMS: Burning Mouth Syndrome; CGI-E: Clinical Global Impression Efficacy, CGI-I- Clinical Global Impression-Improvement; CGI-S: Clinical Global Impression-Severity; PGB: Pregabalin; SSRI: Selective Serotonin Reuptake Inhibitor; SNRI: Dual Serotonin and Norepinephrine Reuptake Inhibitor; VO: Vortioxetine.

**Table 6:** Numbers and types of Adverse Events reported by BMS patients treated with vortioxetine and pregabalin (group A) and an SSRI or SNRI and pregabalin (group B).

**Adverse Events**	**GROUP A** 136 (67.3)	**GROUP B (SSRI/SNRI + PGB) Frequency (%) 67 (32.7)**	***P*-value**
Yes	12(8.8)	14(20.8)	0.024
1 Adverse event More adverse events	8 (5.8) 4 (2.9)	0 (0) 14 (20.8)	0.055 <0.001**
Nausea	4 (2.94)	2 (2.99)	1.000
Abdominal pain	4 (2.94)	0 (0)	0.305
Costipation	2 (1.47)	2 (2.99)	0.600
Dry Mouth	0 (0)	2 (2.99)	0.109
Dizziness	2 (1.47)	1 (1.49)	1.000
QTC prolongation	0 (0)	2 (2.99)	0.109
Elevated serum Prolactine	0 (0)	2 (2.99)	0.109
Somnolence	2 (1.47)	2 (2.99)	0.600
Weight gain	1 (0.74)	2 (2.99)	0.254
Appetit stimulant	1 (0.74)	2 (2.99)	0.254
Sexual dysfunction	0 (0)	2 (2.99)	0.109
Vivid dreams	0 (0)	1 (1.49)	0.330

**Note**: A significant difference between the percentages was measured by the Pearson Chi-Square test. When one or more cells contain a frequency less than 5 then the Fisher Exact Test was used. **Significant with Bonferroni correction 0.004. **Abbreviations**: BMS: Burning Mouth Syndrome; PGB: Pregabalin; SSRI: Selective Serotonin Reuptake Inhibitor; SNRI: Dual Serotonin and Norepinephrine Reuptake Inhibitor; VO: Vortioxetine.

**Table 7:** Features selection predicting clinical response applying forward stepwise logistic regression.

**Clinical Response**	**Beta (SE)**	**OR**	***P*-value**
**Smoker**	1.03	2.79	0.006**
**Physical Activity**	1.51	4.51	0.007**
**Angiotensin II receptor antagonists (ARBs)**	1.02	2.79	0.015*
**Paroxetine**	1.66	5.24	0.046*
**QTC value**	0.02	1.02	0.016*
**Constant**	-9.10	0.00	0.016

**Note**: SE are the standard errors of the beta estimates. OR are the odds ratio. The *p*-values were obtained from the hypothesis test on regression coefficients. *Moderately significant .01 < *p*-value ≤ .05 **Strongly significant *p*-value ≤ .01.

**Table 8:** Multivariate logistic regression analysis in the two groups predicting the clinical response.

**Predictors of Clinical Response in Group A (VO + PGB)**	**Model 1**		**Model 2**	
	***OR***	***P-value***	***OR***	***P-value***
**Age**	0.98	0.400	0.98	0.262
**Gender: Male**	1.30	0.551	1.03	0.951
**Years of education**	1.00	0.943	1.01	0.771
**Marital status: Married**	0.73	0.459	0.76	0.537
**Job: Occupied**	0.72	0.517	0.69	0.498
**Smoker**	2.71	0.044*	2.80	0.045*
**Alcohol**	0.79	0.653	0.63	0.407
**BMI**	0.97	0.568	0.94	0.330
**Physical activity**	2.99	0.080	4.02	0.038*
**Angiotensin II receptor antagonists (ARBs)**	-	-	3.62	0.021
**QTC value**	-	-	1.02	0.037*
***R^2^ (%)***	6.8	0.193	12.9	0.016*
***R^2^ change (%)***	-	-	6.1	0.004*
**Predictors of Clinical Response in Group B (SSRI/SNRI + PGB)**	**Model 1**		**Model 2**	
	***OR***	***P-value***	***OR***	***P-value***
**Age**	0.99	0.781	0.97	0.324
**Gender: Male**	1.39	0.665	1.51	0.597
**Years of education**	1.10	0.195	1.11	0.191
**Marital status: Married**	0.48	0.302	0.65	0.600
**Job: Occupied**	0.54	0.401	0.46	0.344
**Smoker**	2.26	0.216	2.06	0.264
**Alcohol**	2.05	0.398	2.66	0.264
**BMI**	1.05	0.545	1.04	0.674
**Physical activity**	1.46	0.771	2.62	0.477
**Angiotensin II receptor antagonists (ARBs)**	-	-	4.07	0.119
**Paroxetine**	-	-	9.75	0.019*
**QTC value**	-	-	1.02	0.277
***R^2^ (%)***	6.9	0.712	16.1	0.258
***R^2^ change (%)***	-	-	9.2	0.038*

**Note**: OR are the odds ratio. The *p*-values were obtained from the hypothesis test on regression coefficients. *Moderately significant .01 < *p*-value ≤ .05. **Abbreviations**: PGB: Pregabalin; SSRI: Selective Serotonin Reuptake Inhibitor; SNRI: Dual Serotonin and Norepinephrine Reuptake Inhibitor; VO: Vortioxetine.

## Data Availability

The data that support the findings of this study are available from the corresponding author [F.C.], upon reasonable request.

## LIST OF ABBREVIATIONS

AEs: Adverse Events
ARBs: Angiotensin II Receptor Antagonists
BMI: Body Mass Index
BMS: Burning Mouth Syndrome
CGI: Clinical Global Impression Scale
CGI-E: CGI Efficacy Index
CGI-I: CGI Improvement
CGI-S: CGI Severity of Illness
ECG: Electrocardiograms
GAD: Generalized Anxiety Disorder
HAM-A: Hamilton Rating Scale for Anxiety
HAM-D: Hamilton Rating Scale for Depression
MDD: Major Depressive Disorder
PGB: Pregabalin
SF-MPQ: Short-form McGill Pain Questionnaire
SNRI: Serotonin-Norepinephrine Reuptake Inhibitors
SSRI: Selective Serotonin Reuptake Inhibitors
VAS: Visual Analog Scale
VO: Vortioxetine
